# Size-Dependent Magnetic and Magneto-Optical Properties of Bi-Doped Yttrium Iron Garnet Nanopowders

**DOI:** 10.1186/s11671-022-03709-0

**Published:** 2022-08-04

**Authors:** Aleksandr Spivakov, Chun-Rong Lin, Chang-Yen Tsai, Ying-Zhen Chen

**Affiliations:** Department of Applied Physics, National Pingtung University, No. 4-18 Minsheng Rd., Pingtung County, 90003 Taiwan

**Keywords:** Bi-YIG, Nanopowders, MCD spectroscopy, Magnetic properties, Combustion method

## Abstract

Bi-doped yttrium iron garnet nanopowders were successfully synthesized by a combustion method at different synthesis conditions, and the evolution of their structural, magnetic, and magneto-optical properties has been studied by various methods. X-ray diffraction analysis revealed that crystallite size increases with increase as in annealing time (*t*_A_) well as in annealing temperature (*T*_A_) and varied from 15.2 nm (*T*_*A*_ = 650 °C, *t*_*A*_ = 0.5 h) to 44.5 nm (*T*_*A*_ = 800 °C, *t*_*A*_ = 12 h). The magnetic hysteresis loops exhibit behavior characteristic of soft magnetic materials; herewith, the saturation magnetization demonstrates a growing trend with increasing crystallite size (*D*). The behavior of the coercivity indicates that, at room temperature, the transition between single-domain and multidomain states occurs at *D* = 35.3 nm. It was found that the size effect in the MCD spectra is clearly observed for the samples with crystallite sizes less than 42.2 nm for an intersublattice charge-transfer transition and a crystal-field tetrahedral transition. The influence of cation redistribution on the observed changes has been discussed.

## Introduction

Yttrium iron garnet (YIG) is a ferrimagnetic material that has attracted intense research attention for a long time [1–3]. Due to its chemical stability and unique magnetic, magneto-optical, electromagnetic properties [4–6], YIG and substituted YIG have found practical applications in various fields [7–10]. YIG crystallizes in a cubic centrosymmetric structure and belongs to space group Ia3d [11]. In this structure, Fe^3+^ ions are distributed between octahedral [16a] and tetrahedral (24d) sites, while Y^3+^ ions occupy dodecahedral {24c} sites [1, 12] and the structure can be represented by the general formula $\left\{ {Y_{3}^{3 + } } \right\}\left[ {{\text{Fe}}_{2}^{3 + } } \right]\left( {{\text{Fe}}_{3}^{3 + } } \right){\text{O}}_{12}$. Additional interest in the study of YIG is associated with the fact that the properties can be tailored significantly by dopant substitutions [13–17]. As has been reported, the magnetic and optical properties of YIG can be tailored by doping with different cations, such as dysprosium [16, 18], aluminum [10, 19], gadolinium [20, 21], lanthanum [22, 23], and cerium [17, 24]. Among various substituted YIG, Bi-doped yttrium iron garnets, where Bi^3+^ ions replace Y^3+^ ions in dodecahedral sites [25], has stimulated great interest for a long time [26, 27] since it has high transparency at short wavelengths and large Faraday rotation [28, 29]. Due to these properties, Bi-YIG is one of the most promising materials for magneto-optical devices and found practical applications in magneto-optical display devices, magneto-optical disks, optical isolators, magneto-optical microscopy, electric current sensors, and so on [30–35]. In addition to the effect on magneto-optical properties, it has also been reported in the literature on the occurrence of multiferroic properties in Bi-modified YIG [29] and discussed the effect of bismuth on the magnetic [25, 34] and electric [36, 37] properties. However, most of the works on Bi-YIG have been focused on the effect of concentration dependence on physicochemical properties, while information on the effect of synthesis conditions, which can have a significant effect on the properties, is limited.

In this work, Y_1.3_Bi_0.9_Fe_5_O_12_ (Y_1.4_Bi_0.9_Fe_5_O_12_) nanopowders were synthesized by a combustion method at temperatures ranging from 650 to 800 °C and various annealing times for the samples obtained at 650 °C. The effect of the synthesis conditions, which is not related to changes in the concentration of bismuth, on the structural, magnetic, and magneto-optical properties was studied by various methods.

## Methods

### Synthesis of Bi-Doped YIG Nanopowders

Magnetic nanopowders of Bi-YIG were obtained by combustion method using standard complex precursors at various annealing temperatures and annealing time. In a typical process, 0.45 g of yttrium oxide (Y_2_O_3_, Sigma-Aldrich, St. Louis, USA) was first dissolved in 2 ml nitric acid (EMSURE® for Merck Millipore, USA). Then, 8.3 g of iron (III) nitrate nonahydrate (Fe(NO_3_)_3_, Sigma-Aldrich, St. Louis, USA), 3.4 g of bismuth(III) nitrate pentahydrate (Bi(NO_3_)_3_, Sigma-Aldrich, St. Louis, USA) and yttrium oxide in nitric acid were dissolved in a solution of citric acid (1.3 g), glycine (0.35 g) (Acros Organics (Thermo Fisher Scientific) Waltham, USA), DI water. The obtained solution was then heated to 130 °C under magnetic stirring for 30 min to form the gel. When the solution became homogeneous, the temperature was raised to 200 °C for 10 min. The gel ignited spontaneously and formed an aggregate of loose powders. The resulting product was placed in an oven heated to 200 °C and then annealed at ambient conditions at temperatures (*T*_*A*_) ranging from 650 to 800 °C and various annealing times (*t*_*A*_) (for the samples annealed at 650 °C).

### Characterization

The structural analysis of the obtained samples was carried out by X-ray powder diffraction (XRD) using a Shimadzu XRD-6000 diffractometer (Bruker Corp., Billerica, USA) (Cu Ka radiation, 40 kV, 30 mA, *λ* = 1.5406 Å). The particles’ morphology was characterized by scanning electron microscopy (SEM) using Hitachi S-3000 N scanning electron microscope (Hitachi High-Tech Corp., Japan, Tokyo) and transmission electron microscopy (TEM) using the JEOL JEM-1230 transmission electron microscope (JEOL Ltd., Japan, Tokyo) operated at an accelerating voltage of 120 kV. The ICP-MS analysis was carried out using a high-resolution ICP-MS system Thermo Scientific ELEMENT XR (Thermo Fisher Scientific, Waltham, USA). The magnetic properties of the synthesized samples were examined via a vibrating sample magnetometer Lakeshore 7400 series VSM (Lake Shore Cryotronics Inc., Westerville, USA) in the applied field of H = ± 10 kOe. Magnetic circular dichroism (MCD) spectra were recorded in a magnetic field of 8 kOe with a JASCO J-820 spectropolarimeter (JASCO Inc., Mary's Court Easton, USA) equipped with an electromagnet GMW Associates 5403. All measurements were carried out under ambient conditions.

## Results and Discussion

### Structural Characterization

**Fig. 1:**
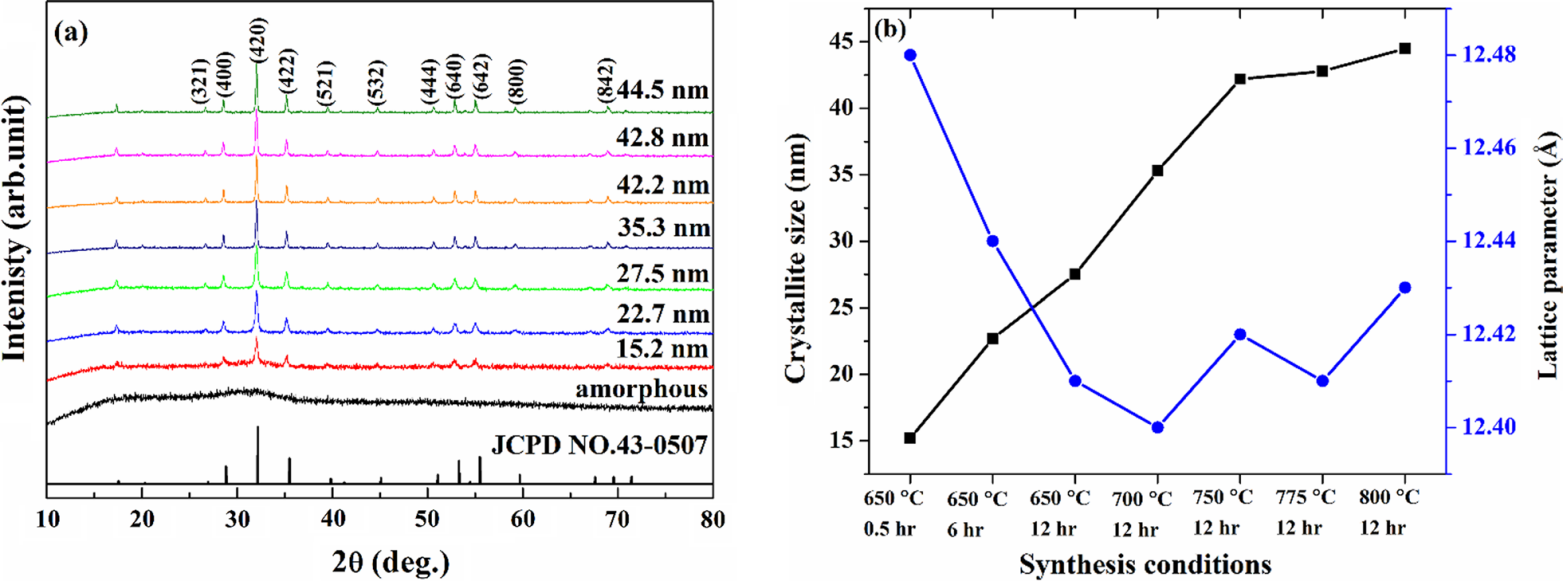
**a** XRD patterns of the Bi-doped YIG nanopowder synthesized at various conditions and, **b** variation of crystallite size and lattice parameter with the change of the synthesis conditions

As can be seen from Fig. 1a, the diffraction peaks are consistent well with the standard JCPDS card # 43-0507 of Y_3_Fe_5_O_12_ with the cubic garnet structure and do not contain features of any impurities [34, 37]. In addition, the obtained patterns demonstrate that with an increase in both the annealing time and the reaction temperature, peaks become narrow, and their intensity increases, indicating an increase in the average crystallite size and better crystallinity of the samples with the increase in annealing temperature and time. As can be seen from Fig. 1a, the diffraction peaks (420) and (422) are shifted toward lower 2θ values compared with pure Y_3_Fe_5_O_12_, which indicates that Bi atoms were incorporated into the garnet structure [38].

The average crystallite size (*D*) of the obtained samples was calculated using the Scherrer Eq. (1) based on the FWHM of the most intense peak (420), and obtained values are shown in Fig. 1b. As can be seen from the figure, the average crystallite size increases with an increase in both the annealing time and synthesize temperature, which demonstrates that in the synthesis process, the crystallite size can be controlled by the change of both these parameters.

2 $$D = \frac{0.89\lambda }{{\beta \cos \theta }} \left( 1 \right);\quad a = \frac{\lambda }{2\sin \theta }\sqrt {h^{2} + k^{2} + l^{2} }$$

where *λ* is the radiation wavelength (0.15406 nm for Cu Kα); *β* is the line broadening of a diffraction peak at angle *θ*; and (hkl) are the Miller indices.

Based on Bragg's law and the d-spacing equation for a cubic structure, the lattice parameter of the samples was calculated in accordance with relations (2) for the peaks (400), (420), (422), (640), (642), and (842). The obtained values were then extrapolated to ${\text{cos}}^{2} \theta = 0$ according to Bradley and Jay’s method [39] to minimize systematic errors. A variation of the lattice parameter with the change in the synthesis conditions and, consequently, with a change in the average crystallite size is shown in Fig. 1b. The obtained values of the lattice parameter for all samples are larger than reported in the literature for pure yttrium iron garnet nanoparticles [40–42], which is related to the larger ionic radius of Bi^3+^ (1.17 Å) compared with the ionic radius of Y^3+^ (1.019 Å) for the eightfold coordination [43]. Additionally, these values agree well with previous results obtained for Bi_*x*_Y_3−*x*_Fe_5_O_12_ particles with *x* ~ 1 [44, 45]. The lattice parameter demonstrates a decreasing trend (for particles with *D* ≤ 35.3 nm), which may be associated with the influence of the size effect on the crystal lattice. According to P. Ayyub et al. [46], a decrease in lattice constant with an increase in particle size is related to unpaired electronic orbitals on the outer surface of particles, which repel each other, leading to an increase in the equilibrium lattice constant compared to the bulk crystal. An increase in the lattice parameter for samples with *D* > 35.3 nm is attributed to the influence of the redistribution of cations, which increases with an increase in the annealing temperature [47], which is consistent with the obtained results.

To determine the actual compositions of the obtained samples, ICP-MS analysis was performed, and the results are summarized in Table 1. The results obtained demonstrate that the bismuth content in the samples remains constant with a change in the synthesis conditions; therefore, the changes in the structural, magnetic, and magneto-optical properties cannot be associated with changes in the Bi content in the synthesized samples.

**Table 1:** The actual compositions of Bi-doped YIG nanopowder synthesized at different synthesis conditions obtained from ICP-MS analysis

Synthesis conditions	Actual composition
*T*_*A*_ = 650 °C, *t*_*A*_ = 0.5 h	Y_1.3_Bi_0.9_Fe_5_O_12_
*T*_*A*_ = 650 °C, *t*_*A*_ = 6 h	Y_1.4_Bi_0.9_Fe_5_O_12_
*T*_*A*_ = 650 °C, *t*_*A*_ = 12 h	Y_1.3_Bi_0.9_Fe_5_O_12_
*T*_*A*_ = 700 °C, *t*_*A*_ = 12 h	Y_1.3_Bi_0.9_Fe_5_O_12_
*T*_*A*_ = 750 °C, *t*_*A*_ = 12 h	Y_1.3_Bi_0.9_Fe_5_O_12_
*T*_*A*_ = 775 °C, *t*_*A*_ = 12 h	Y_1.4_Bi_0.9_Fe_5_O_12_
*T*_*A*_ = 800 °C, *t*_*A*_ = 12 h	Y_1.4_Bi_0.9_Fe_5_O_12_

**Fig. 2:**
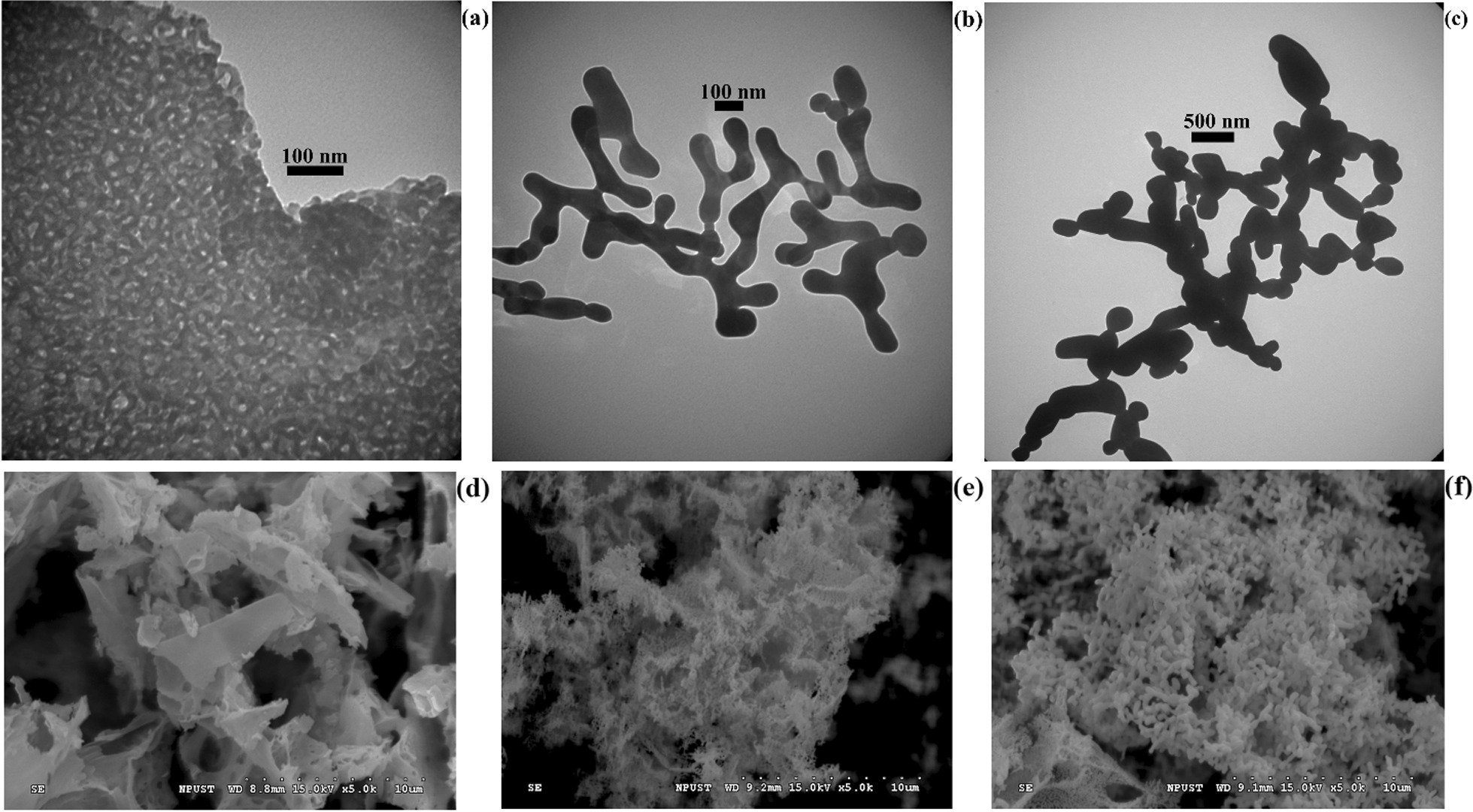
TEM and SEM images of the samples synthesized at the following conditions: **a** and **d**—*T*_*A*_ = 650 °C; *t*_*A*_ = 0.5 h, **b** and **e**—*T*_*A*_ = 700 °C; *t*_*A*_ = 12 h, **c** and **f**—*T*_*A*_ = 800 °C; *t*_*A*_ = 12 h

The TEM images of the samples obtained at *T*_*A*_ = 650 °C; *t*_*A*_ = 0.5 h., *T*_*A*_ = 700 °C; *t*_*A*_ = 12 h., and *T*_*A*_ = 800 °C; *t*_*A*_ = 12 h. are shown in Fig. 2a–c, and the corresponding SEM micrographs are presented in Fig. 2d–f.

As can be seen from Fig. 2a, d, at the annealing temperature of 650 °C, grains with a flake-like structure are formed, while the nanoparticles demonstrate a high level of agglomeration and form a network structure. However, with the increase in the annealing temperature, roughly spherical grains are formed, which merge as a result of the interaction of long-range magnetic dipole–dipoles between the particles. In these samples, nanoparticles partially merge and form elongated structures. Due to the high agglomeration of the nanoparticles and their irregular shapes, it was not possible to determine their size with a sufficient degree of accuracy based on the TEM data; however, it can be concluded from the figures that the increase in the annealing temperature leads to an increase in particle size, which agrees with the results of the XRD analysis.

### Magnetic Measurements

**Fig. 3:**
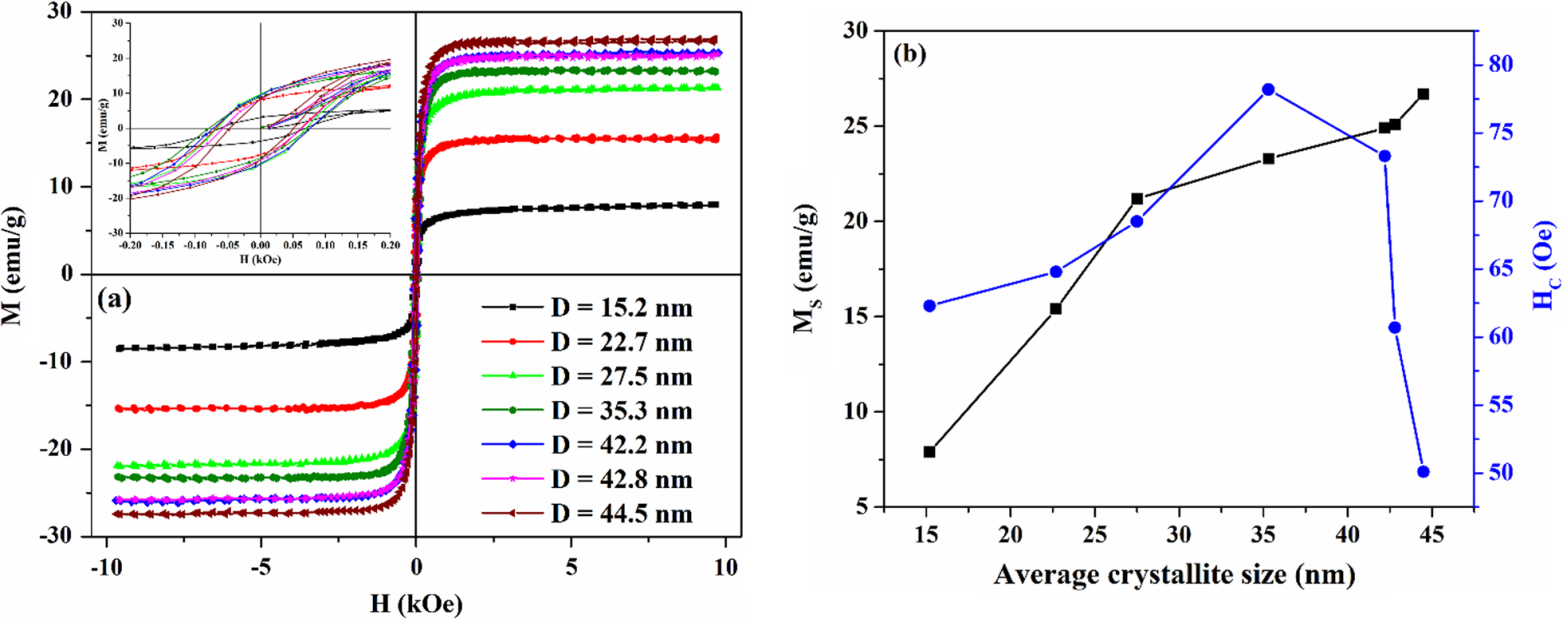
**a** Magnetic hysteresis loops of the samples obtained at various annealing temperatures and annealing times; **b** dependence of saturation magnetization and coercivity on crystallite size. The inset shows the hysteresis loops on an enlarged scale

The field-dependent magnetization curves measured at room temperature of the samples are shown in Fig. 3a, b demonstrating the dependence of saturation magnetization (*M*_*S*_) and coercivity (*H*_*C*_) on crystallite size. The values of magnetic parameters obtained from the curves are listed in Table 2. It can be seen from Fig. 3a and Table 2 that the values of coercivity and remanence magnetization are low, which is a characteristic of soft magnetic materials. The obtained values of *M*_*S*_ for the synthesized samples are in good agreement with values of *M*_*S*_ reported in the literature for particles with comparable crystallite sizes, which were synthesized by various methods, such as different sol–gel technics [25, 34, 48, 49], low-temperature co-fired method [50], and solid-state reaction [36].

**Table 2:** Magnetic parameters of the Bi-doped YIG nanopowders synthesized at different reaction conditions

Synthesis conditions	650 °C, 0.5 h	650 °C, 6 h	650 °C, 12 h	700 °C, 12 h	750 °C, 12 h	775 °C, 12 h	800 °C, 12 h
*D*, nm	15.2	22.7	27.5	35.3	42.2	42.8	44.5
*M*_*S*_, emu/g	7.9	15.4	21.2	23.3	24.9	25.1	26.7
*M*_*R*_, emu/g	3.4	8.1	10.2	8.7	9.8	8.9	8.7
*M*_*R*_/*M*_*S*_	0.43	0.53	0.48	0.37	0.39	0.35	0.33
*H*_*C*_, Oe	62.3	64.8	68.5	78.2	73.3	60.7	50.1

*M*_*S*_, saturation magnetization; *M*_*R*_, remanent magnetization; *H*_*C*_, coercivity; and *M*_*R*_/*M*_*S*_, squareness ratio

The obtained results demonstrate (Fig. 3b) that the saturation magnetization increases with an increase in crystallite size. The decrease in saturation magnetization of the samples with decreasing crystallite size can be explained by the “dead” layer theory [51, 52]. It is assumed that the outer layer of the magnetic particles contains canted or disordered spins, which result from broken exchange bonds [53]. These spins prevent the core spins to align along the field direction, resulting in a decrease in the saturation magnetization with a decrease in particle size. Another reason affecting the decrease in *M*_*S*_ is a redistribution of cations between sublattices. The net magnetization of garnets, according to Neel’s theory [54], is proportional to the difference between the magnetic moment of tetrahedral *M*_(*d*)_, octahedral *M*_[*a*]_, and dodecahedral *M*_{*c*}_ sublattices and is given by: $M_{S} = \left| {M_{\left( d \right)} - M_{\left[ a \right]} } \right| - M_{{\left\{ c \right\}}}$, where *M*_{*c*}_ = 0, since dodecahedral sites are occupied by non-magnetic Y^3+^ ions. It was suggested that [55] a decrease in particle size leads to a decrease in surface energy, and the energy becomes insufficient to retain the preferred distribution of cations in the spinel structure, and cation redistribution may occur, which will affect the net magnetization [56, 57]. As will be shown below by the MCD spectroscopy, some of the Fe^3+^ ions migrate from the tetrahedral sites, and their number increases with decreasing crystallite size in the range of 15.2 ≤ *D* ≤ 35.3 nm, which can be due to the fact that surface energy decreasing more and more with a gradual decrease in particle size. The redistribution of cations from the tetrahedral sublattice will lead to a weakening of the tetrahedral magnetic moment *M*_(*d*)_ and, consequently, the net magnetization.

As can be seen from Table 2, the coercivity first increases from 62.3 Oe (*D* = 15.2 nm) to 78.2 Oe (*D* = 35.3 nm) with the corresponding increase in the average crystallite size, but with a further increase in *D*, the coercivity decreases to 50.1 Oe (*D* = 44.5 nm). Such behavior is related to the fact that the coercivity of magnetic nanoparticles is closely related to their size by the Stoner–Wohlfarth single-domain theory [58]. In the single- and multidomain regions, the size dependence of coercivity can be approximately expressed by the following equations [59, 60]:

$$H_{C} = g - {\raise0.7ex\hbox{$h$} \!\mathord{\left/ {\vphantom {h {D^{3/2} }}}\right.\kern-\nulldelimiterspace} \!\lower0.7ex\hbox{${D^{3/2} }$}}\left( {{\text{single }}\,{\text{domain}}\,{\text{region}}} \right); H_{C} = a + b/D \left( {{\text{multidomain }}\,{\text{region}}} \right),$$

where *a*, *b*, *g*, *h* are constants, and *D* is particle size.

Thus, coercivity should have a maximum value, *D*_cr_, at which the single-domain structure splits into several domains in order to reduce the large magnetization energy, which leads to a decrease in coercivity. As follows from the obtained results, for the synthesized Bi-doped YIG samples, *D*_cr_ = 35.3 nm.

### Results of MCD Spectroscopy

**Fig. 4:**
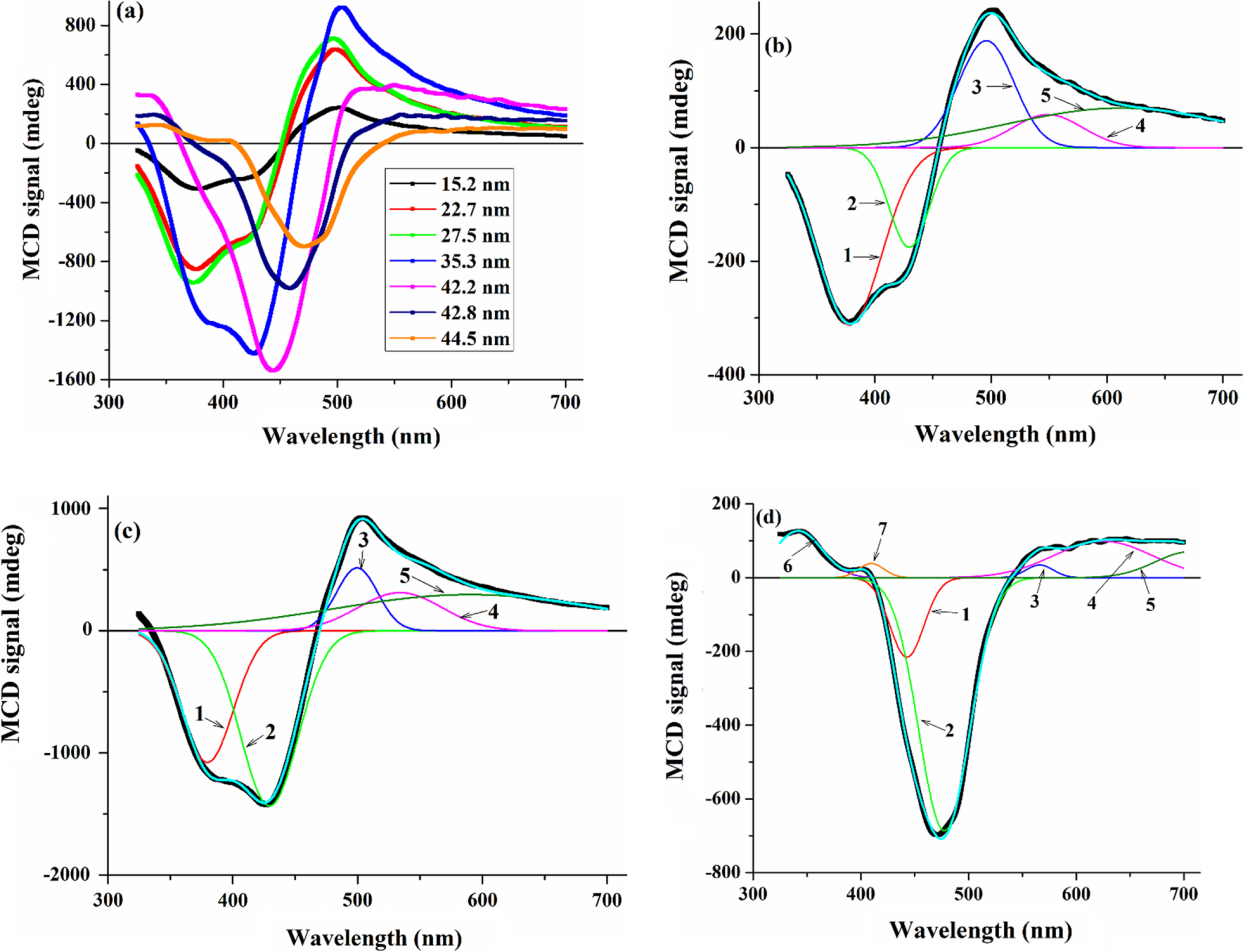
**a** MCD spectra of the Bi-doped YIG nanopowder and the best Gaussian fitting for the samples with **b** D = 15.2 nm, **c** 35.3 nm, **d** 44.5 nm

The MCD spectra of all synthesized samples and Gaussian band fitting of the selected spectra are shown in Fig. 4.

Two main features can be distinguished in the obtained spectra: negative and positive, and their evolution is closely related to crystallite size. The negative feature is present in all samples and undergoes a redshift with an increase in *D*. At the same time, the intensity of the positive feature first increases, but when the crystallite size exceeds 35.3 nm, this feature almost disappears; however, a new positive feature appears at lower wavelengths. To analyze the MCD data, we have decomposed them into a set of Gaussian components. In order to obtain a good agreement between the sum of the components and the experimental spectra, five components were necessary for the samples with *D* ≤ 35 nm, six for the samples with *D* = 42.2 and 42.8 nm, and seven for the sample with *D* = 44.5 nm (Table3).

**Table 3:** Peaks position (*E*, in eV and nm) and peaks intensity (*I*, arb. unit) of the Gaussian components obtained from MCD spectra analysis

Component	*D* = 15.2 nm			*D* = 22.7 nm			*D* = 27.5 nm			*D* = 35.3 nm		
	*E*		*I*	*E*		*I*	*E*		*I*	*E*		*I*
1	3.28	378	− 312.2	3.29	377	− 861	3.32	374	− 950.4	3.26	380	− 1076.4
2	2.88	430	− 175.3	2.89	429	− 465.1	2.9	428	− 495.5	2.88	430	− 1432.9
3	2.5	496	188.4	2.51	493	481.8	2.54	489	476.6	2.48	500	514.4
4	2.26	549	58.1	2.29	541	172.8	2.35	528	221.4	2.32	534	312
5	2.03	612	69.6	2.05	606	168.2	2.03	612	148.5	2.1	590	296.1
6	–	–	–	–	–	–	–	–	–	–	–	–
7	–	–	–	–	–	–	–	–	–	–	–	–

Component	*D* = 42.2 nm			*D* = 42.8 nm			*D* = 44.5 nm		
	*E*		*I*	*E*		*I*	*E*		*I*
1	3.21	386	− 210.6	3.16	392	− 40.6	2.8	443	− 215.4
2	2.79	445	− 1551.9	2.71	457	− 1009.3	2.59	478	− 685.8
3	2.44	508	212.5	2.35	527	67.2	2.19	566	34.4
4	2.29	541	291.5	2.19	565	150.4	1.98	625	99
5	1.93	641	293.9	1.88	661	170.9	1.76	706	71.1
6	3.7	335	338.7	3.66	339	199.3	3.63	342	127.1
7	–		–	–	–	–	3.02	410	38.4

Based on previous theoretical and experimental studies of YIG and substituted YIG [61–65], the decomposition components have been identified with transitions of different nature. It is assumed that at the energy region ~ 3 to 4.5 eV, the transitions in YIG are related to a mixture of biexciton excitation of Fe^3+^ (simultaneous crystal-field transitions of two neighboring ions) and charge-transfer transitions where the charge is transferred between octahedral and tetrahedral Fe^3+^. Thus, the following peaks can be attributed to this type of transition: peak 1 in all samples, excluding the sample with *D* = 44.5 nm, and peaks numbered 6 and 7 in the samples with *D* = 42.2, 42.8, and 44.5 nm. The position of peak 2 for the samples with 15.2 ≤ *D* ≤ 35.3 is agreed with an intersublattice charge-transfer transition between tetrahedral and octahedral iron ions. All other peaks are associated with crystal-field tetrahedral (CFT) and crystal-field octahedral (CFO) transitions and can be assigned to the following transitions: Peak 3 for the samples with 15.2 ≤ *D* ≤ 35.3 nm can be attributed to components of ^6^A_1_(^6^S) → ^4^E, ^4^A_1_(^4^G) CFT transition; peaks numbered 4 (15.2 ≤ *D* ≤ 42.2 nm) and 3 (*D* = 42.8 nm) can be assigned to the low-energy side of the ^6^A_1_(^6^S) → ^4^T_2_(^4^G) transition or the high-energy side of the ^6^A_1_(^6^S) → ^4^T_1_(^4^G) transition; peak 5 (15.2 ≤ *D* ≤ 42.2), peaks 4 and 5 (*D* = 42.8), and peaks 3 and 4 (*D* = 44.5) are attributed to ^6^A_1_(^6^S) → ^4^T_1_(^4^G) CFT transition, which consists of three positive components; however, only two of these components can be resolved in our spectra for samples with *D* = 42.8 and 44.5 nm, and other samples contain only one component; peak 1 (*D* = 44.5) and peak 2 (*D* = 42.2) correspond to ^6^A_1g_(^6^S) → ^4^T_2g_(^4^D) CFO transition; peak 2 for *D* = 42.8 nm and peak 2 for the sample with *D* = 44.5 nm are related to components of ^6^A_1g_(^6^S) → ^4^E_g_, ^4^A_1g_(^4^G) CFO transition; and peak 5 (*D* = 44.5) is consistent with ^6^A_1g_(^6^S)—^4^T_2g_(^4^G) CFO transition.

The obtained results demonstrate that in the samples with 15.2 ≤ *D* ≤ 35.3 nm, the intensity of peaks 2 and 3 decreases with decreasing crystallite size, which can be explained by the redistribution of cations. Peak 2 is an intersublattice charge-transfer transition between the same ions belonging to different magnetic sublattices, namely (Fe^3+^) → [Fe^3+^]. Thus, a decrease in the intensity of this peak indicates that some of the Fe^3+^ ions migrate from the tetrahedral sublattice, and their number increases with decreasing crystallite size. At the same time, a decrease in the number of ions (Fe^3+^) should lead to a decrease in the intensity of the crystal-field tetrahedral transition (Peak 3 in these samples) [66], which is consistent with the results obtained. Thus, it can be concluded that in the case of the decrease in the size of synthesized nanopowders, a redistribution of cations occurs not only between octahedral and dodecahedral sublattices (as in the case of an increase in the Bi content in the Bi_*x*_Y_3–*x*_Fe_5_O_12_ system [34]) but also, probably, between tetrahedral and dodecahedral sublattices. It is assumed that the transition to the multidomain structure with the subsequent increase in crystallite size causes a significant rearrangement of cations in the crystal lattice, which leads to changes in the structure of the MCD spectra for samples with *D* > 35.3 nm.

## Conclusions

The influence of the synthesis conditions such as annealing time and annealed temperature on the structural, magnetic, and magneto-optical properties of the Bi-doped YIG nanopowders obtained by the combustion method has been studied. The results of the structural analysis revealed the formation of the cubic garnet structure without impurities for all samples; herewith, an increase in both the annealed temperature and annealing time leads to the increase in crystallite size. Magnetic measurements showed that the coercivity first increases and then reaches a maximum value of 78.2 Oe at *D* = 35.3 nm and with a further increase in *D*, the coercivity decreases, which indicates the presence of a transition between a single-domain and multidomain states. The saturation magnetization increases with increasing crystallite size, and the obtained values are in agreement with values reported in the literature for samples with comparable crystallite sizes. The observed change in the saturation magnetization can be explained by the redistribution of cations with an increase in the crystallite size. The MCD spectra of the synthesized samples were decomposed to the sets of Gaussian components to provide a good agreement between the experimental spectra and the sum of the components. The obtained components were identified with transitions of different nature. It was found that the size effect is observed for the samples with 15.2 ≤ *D* ≤ 35.3. In these samples, a (Fe^3+^) → [Fe^3+^] charge-transfer transition and a crystal-field tetrahedral transition tend to decrease with decreasing crystallite size. Such behavior was explained by the migration of Fe^3+^ ions from tetrahedral sites.

## Data Availability

The raw and processed data required to reproduce these findings cannot be shared at this time as the data also form part of an ongoing study. However, some data required to reproduce these results can be provided upon request by email: aleksandr.a.spivakov@gmail.com.
